# Preparation and Characterization of Curcumin Nanoemulgel Utilizing Ultrasonication Technique for Wound Healing: In Vitro, Ex Vivo, and In Vivo Evaluation

**DOI:** 10.3390/gels7040213

**Published:** 2021-11-14

**Authors:** Mohammed S. Algahtani, Mohammad Zaki Ahmad, Ihab Hamed Nourein, Hassan A. Albarqi, Hamad S. Alyami, Mohammad H. Alyami, Abdulsalam A. Alqahtani, Ali Alasiri, Thamer S. Algahtani, Abdul Aleem Mohammed, Javed Ahmad

**Affiliations:** 1 Department of Pharmaceutics, College of Pharmacy, Najran University, Najran 11001, Saudi Arabia; msalqahtane@nu.edu.sa (M.S.A.); mzahmad@nu.edu.sa (M.Z.A.); haalbarqi@nu.edu.sa (H.A.A.); hsalmukalas@nu.edu.sa (H.S.A.); mhalmansour@nu.edu.sa (M.H.A.); aamari@nu.edu.sa (A.A.A.); aalasiri@nu.edu.sa (A.A.); tsalqahtany@nu.edu.sa (T.S.A.); aaleem@nu.edu.sa (A.A.M.); 2 Department of Clinical Laboratory (Histopathology and Cytology), College of Applied Medical Sciences, Najran University, Najran 11001, Saudi Arabia; ihab213@gmail.com

**Keywords:** curcumin, nanoemulsion, ultrasonic emulsification, nanoemulgel, thixotropy, wound healing

## Abstract

Hydrogels being a drug delivery system has great significance particularly for topical application in cutaneous open wound. Its specific physicochemical properties such as non-adhesiveness, moisture retention, exudate absorption, and gas permeability make them ideal as a drug delivery vehicle for wound healing application. Further, curcumin (a natural bioactive) was selected as a therapeutic agent to incorporate into the hydrogel system to design and develop nanogel pharmaceutical products for wound healing. Although, curcumin possesses remarkable anti-inflammatory, antioxidant, and anti-infective activity along with hastening the healing process by acting over the different stages of the wound healing process, but its poor biopharmaceutical (low aqueous solubility and skin penetrability) attributes hamper their therapeutic efficacy for skin applications. The current investigation aimed to develop the curcumin-loaded nanogel system and evaluated to check the improvement in the therapeutic efficacy of curcumin through a nanomedicine-based approach for wound healing activity in Wistar rats. The curcumin was enclosed inside the nanoemulsion system prepared through a high-energy ultrasonic emulsification technique at a minimum concentration of surfactant required to nanoemulsify the curcumin-loaded oil system (Labrafac PG) having droplet size 56.25 ± 0.69 nm with polydispersity index 0.05 ± 0.01 and negatively surface charge with zeta potential −20.26 ± 0.65 mV. It was observed that the impact of Smix (surfactant/co-surfactant mixture) ratio on droplet size of generated nanoemulsion is more pronounced at lower Smix concentration (25%) compared to the higher Smix concentration (30%). The optimized curcumin-loaded nanoemulsion was incorporated into a 0.5% Carbopol^®^ 940 hydrogel system for topical application. The developed curcumin nanoemulgel exhibited thixotropic rheological behavior and a significant (*p* < 0.05) increase in skin penetrability characteristics compared to curcumin dispersed in conventional hydrogel system. The in vivo wound healing efficacy study and histological examination of healed tissue specimen further signify the role of the nanomedicine-based approach to improve the biopharmaceutical attributes of curcumin.

## 1. Introduction

Wounds are physical injuries, leading to an opening or break of the skin [1]. The appropriate healing of wounds is vital for the repair of disrupted anatomical and functional status of the skin. Restoring wounds is one of the most complicated physiological processes that starts from the response to an injury to restoring the function and integrity of damaged tissues [2]. Wound healing involves numerous physiological events as clotting, coagulation, inflammation, and the generation of fresh tissues, which may follow varied timescale from minutes to numerous months or years [3]. Any deviations or delays to the multistage healing process might be the reason for the failure of wound healing and the development of an incomplete healing process [4]. Long-lasting wounds can seriously affect the quality of life and their treatment requires a very high quality of care [5,6]. It may lead to an increase in the risk of morbidity and mortality. This is especially true for people who suffer from vascular diseases and diabetes mellitus [4]. Therefore, it is essential to optimize a wound healing method that can minimize tissue damage and accelerate the wound healing time. Wound healing agents are available in different routes of administration including oral and parenteral; however, the systemic drug administration of these agents can cause undesirable systemic side effects. Hence, a topical drug delivery system is an attractive approach that can improve the wound healing process and minimize systemic side effects.

Curcumin (o-methoxy phenol derivative compound) is a hydrophobic polyphenol derived from the rhizome of the herb *Curcuma longa*, belonging to the family Zingiberaceae. Curcumin has been used traditionally for several diseases due to its wide spectrum of biological and pharmacological activities [7]. It has been reported to exhibit multifunctional properties, including antioxidant, anti-inflammatory, anti-microbial, and anti-carcinogenic activities [8,9]. Furthermore, topical application of curcumin has been shown to improve the wound healing process and prevent oxidative damage to tissues [8,9]. Additionally, it has been reported that curcumin enhances the production of granulation in body tissue, including a greater amount of cellular content and new vascularization, along with increasing the process of re-epithelialization of wounds [9]. Despite the significant pharmaceutical value of curcumin, the therapeutic applications of curcumin are limited due to its poor aqueous solubility, poor absorption, rapid metabolism, rapid systemic elimination, and low stability [5]. Moreover, the poor aqueous solubility of curcumin limits its skin permeation through the stratum corneum (SC), and poses a major limitation on its topical application in wound healing.

To solve this, there is a need for a topical delivery formulation that enhances the water solubility of curcumin, which promotes its permeation through the skin. Recent studies reported in which drugs are loaded inside the core of nanoemulsion (NE) have contributed to increasing the drug permeation through the skin due to high penetrability through the subcutaneous barrier [10,11]. For example, Algahtani et al. designed a NE formulation to deliver curcumin utilizing the low energy technique for psoriasis treatment [12]. The formulation then was loaded into Carbopol® gel to form a curcumin nanoemulgel (NEG). This formulation has shown faster healing for the psoriatic symptoms when compared to the curcumin suspension and betamethasone-17-valerate, which is commonly used for the treatment of psoriasis. The NE-based formulation for topical delivery of curcumin increases drug concentrations at the area treated, drug-loading capacity, improves skin penetrability, and prolongs the amount of drug release and those properties helpful for successful wound healing [13,14]. The poorly water-soluble curcumin is encapsulated into a lipophilic environment of an oil droplet of nano dimension, which offers high drug-loading, and is stabilized through an optimized amount of surfactant/co-surfactant mixture (Smix) along with an aqueous phase [14].

The low-energy technique used for the production of NE consumes a high amount of surfactants and co-surfactants that might irritate when applied to an open wound. Here, the curcumin NE formulated utilizing the high energy technique through ultrasonication of the formulation that reduces the needed amount of surfactants [15]. Different NE compositions were evaluated to select the optimum formulation. Due to low retention of NE system at the site of application because of low viscosity of NE system [14] and because of the inconvenient application of such formulation, the optimized NE system was dispersed into a hydrogel system to make NEG. Hydrogels being a drug delivery system has great significance particularly for topical application in cutaneous open wound. Its specific physicochemical properties such as non-adhesiveness, moisture retention, exudate absorption, and gas permeability make them ideal as a drug delivery vehicle for wound healing application [16]. In vitro evaluation has been applied to the developed NE system including the measurement of the droplet size, size distribution, zeta potential, and drug release. Ex vivo drug permeation and deposition through rat skin were also evaluated in a developed NEG system. The in vivo wound healing property of the developed formulation was evaluated on rat model and the speed of healing was compared to silver sulfadiazine cream, a common wound healing formulation available in the market.

## 2. Results and Discussion

### 2.1. Screening of NE Components

The Labrafac PG was selected as the oil phase of the NE system based on maximum curcumin solubility in it as previously investigated in our lab [12]. The Tween 80 and PEG 400 are selected as favorable Smix (surfactant and co-surfactant combination) phases based on the desirability of curcumin solubility. Further, it is a widely accepted Smix combination utilized in pharmaceutical products and compatible with the skin. Although the solubility of curcumin in Labrafac PG, Tween 80, and PEG 400 is desirable to achieve sufficient drug loading (10 mg/mL) in the NE system, indeed these components should be helpful to sufficiently nanoemulsify at a low concentration of Smix for the better suitability in wound healing application. The results of emulsification efficiency of Smix phase at different ratios (1:1, 2:1, 3:1, and 4:1) and corresponding %T values were determined and illustrated in Supplementary Figure S1.

Therefore, to design NE system of <100 nm (size dimension desirable for topical application) utilizing Labrafac PG, Tween 80, and PEG 400 as formulation components at a minimum concentration of surfactant and maximum concentration of oil phase, a high-energy ultrasonication technique was adopted. The low-energy self-emulsification technique is a commonly preferred method to prepare an NE system of <100 nm for topical application at a maximum concentration of surfactant and minimum concentration of oil phase.

### 2.2. Preparation of NE through Ultrasonication

Curcumin-loaded NE system was prepared by high-energy ultrasonication technique [17,18] utilizing Labrafac PG as oil phase, Tween 80 as a surfactant, and PEG 400 as co-surfactant at variable % composition shown in Table 1. The % composition of different formulations shown in Table 1 has been selected based on the curcumin loading in each formulation being 10 mg/mL. Therefore, the maximum possible concentration of the oil phase (20%) was selected and it remains constant in each formulation to achieve uniform drug loading (10 mg/mL) in each NE system.

In the beginning, the uniform dispersion system was obtained by mixing the 1% *w*/*w* (10 mg/g) of curcumin in the mixture of the oil phase and Smix phase (at a ratio of 1:1 and 2:1 with variable concentration) through vortex mixture. After that, the aqueous phase was added with continuous vortexing for 1 min [15]. The uniform dispersion system obtained was further ultrasonicated (Ultrasonic Homogenizer, FS-300N, Zhejiang, China) in a water bath for varying time duration (3, and 5 min) at a constant ultrasonication amplitude of 40% [17,19]. The impact of the ultrasonication process and composition variables on droplet size and PDI of the generated NE system loaded with curcumin was evaluated as shown in Table 1.

#### 2.2.1. Influence of Smix Ratio and Smix Concentration on Droplet Size

**Figure 1:**
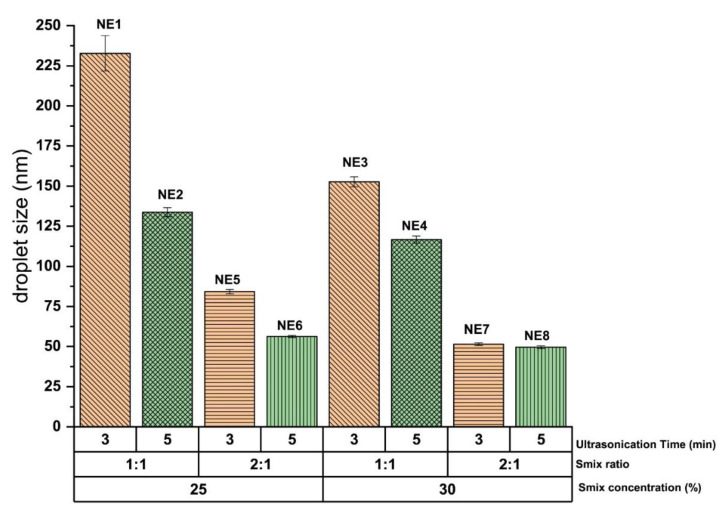
Impact of the process variable (ultrasonication time 3 and 5 min) and composition variables (Smix ratio and % concentration) on droplet size of NE system generated through high-energy ultrasonication technique at Smix concentration 25% (Smix ratio 1:1 and 2:1) and Smix 30% (Smix ratio 1:1 and 2:1). Colors and shaded bars represent variability in formulation compositions and process conditions.

The droplet size of the curcumin-loaded NE system obtained through the high-energy ultrasonication technique was greatly influenced by the ratio of Smix phase present as formulation components of the NE system. It was observed that the NE system composed of Smix ratio 2:1 (at constant Smix concentration and ultrasonication time) significantly (*p* < 0.05) reduced the droplet size compared to the NE system composed of Smix ratio 1:1 (at constant Smix concentration and ultrasonication time). This influence is shown in Figure 1 at Smix concentration of 25 and 30% respectively. This influence of Smix ratio on droplet size is more pronounced at lower concentration (25%) of Smix compared to the higher Smix concentration (30%).

#### 2.2.2. Influence of Ultrasonication Time on Droplet Size

The droplet size of the curcumin-loaded NE system obtained through the high-energy ultrasonication technique was also greatly influenced by the ultrasonication time. It was observed that 5 min ultrasonication significantly (*p* < 0.05) reduces the droplet size of the NE system of constant concentration and the ratio of Smix compared to the 3 min ultrasonication. This influence is shown in Figure 1 at Smix concentration 25 and 30%, respectively. This influence of ultrasonication time on droplet size is more pronounced at 25% concentration of Smix compared to the 30% of Smix concentration. The NE system of comparatively higher Smix concentration is sufficient to reduce the droplet size up to a certain extent compared to the NE system of low Smix concentration. Therefore, the influence of ultrasonication time on droplet size is more pronounced in the NE system having low Smix concentration compared to the NE system of higher Smix concentration.

The Smix ratio, the concentration of Smix phase, and ultrasonication time have no remarkable effect on PDI. It is might be correlated to the HLB value of Labrafac PG (HLB value 2) compared to the natural origin fixed oils (such as black seed oil, sesame oil, and castor oil, etc.). It was observed in the previous investigation of our lab that the impact of Smix ratio, the concentration of Smix phase, and ultrasonication time on PDI of generated NE system through high-energy ultrasonication technique was significantly evident in the case of the NE system of black seed oil [17].

Out of the eight formulations developed, four formulations (NE5, NE6, NE7, and NE8) show droplet size <100 nm (Table 1). The mean droplet size of these four selected NE systems varies between 84.23 ± 1.33 to 49.61 ± 0.53 nm, and the PDI varies between 0.23 ± 0.05 to 0.10 ± 0.01. It is very interesting to observe that curcumin NE prepared through high energy ultrasonic emulsification technique can achieve the droplet size <100 nm at a significantly (*p* < 0.05) lower concentration of Smix compared to the curcumin NE prepared through low energy spontaneous emulsification technique. It is widely seen that NE prepared through low energy spontaneous emulsification technique commonly required two or more than two times of Smix concentration relative to oil concentration in the NE composition to achieve the droplet size <100 nm [12,20,21,22,23], while NE prepared through high energy ultrasonic emulsification technique able to achieve the same droplet size even at Smix concentration less than 1.5 times relative to oil concentration [15]. Indeed, droplet size of NE can also be influenced by the type of oil, surfactant, and co-surfactant used in its preparation in addition to the type of emulsification technique (high-energy/low-energy) utilized for the formation of NE system.

Those four curcumin-loaded NE systems having droplet size <100 nm were selected for further characterization such as thermodynamic stability, viscosity, and % drug content.

### 2.3. Characterization of NE

#### 2.3.1. Analysis of Thermodynamic Stability and Zeta Potential

Thermodynamic stress testing was performed to find out the presence of any metastable NE in the screened formulations. It is determined by the exchange of free energy between the system’s milieu and the system itself [24]. Table 2 shows the results of the thermodynamic stability investigation of the selected formulations (NE5, NE6, NE7, and NE8). The four NE formulations tested were stable under the heat-cooling cycle, freeze-thaw cycle, and centrifugation study. This stability may be related to the high zeta potential of the screened NE formulations (NE5, NE6, NE7, and NE8), which ranges between −15.96 ± 0.55 mV and −20.26 ± 0.65 mV (Table 2). The magnitude of the surface charges has a direct relationship to the stability of the NE system. The high repulsive force between NE droplets minimizes any chances of coalescence and prevents the possibility of physical instability of generated NE system [25].

#### 2.3.2. Viscosity

The viscosity of four screened NE system (NE5, NE6, NE7, and NE8) were tested at room temperature through rotational viscometer. The NE5 has the lowest viscosity value with 83.74 ± 1.92 mPas, while NE8 has the highest viscosity value with 89.82 ± 1.27 mPas (Table 2). This variation in viscosity value is related to the higher concentration of surfactant (Tween 80) in the NE8 formulation system compared to the NE5 formulation system.

#### 2.3.3. Analysis of Drug Content

The UV-visible spectrophotometric analysis was carried out to determine the curcumin content in NE systems [26,27] and it was used here to quantify the % content of the curcumin in the selected four NE formulations (NE5, NE6, NE7, and NE8). The % drug content in curcumin NE formulations are between 98.86 ± 0.58 to 99.23 ± 0.28 % (Table 2), which indicates that the curcumin is almost completely encapsulated into oil droplets of Labrafac PG-based NE system.

All of the selected formulations have passed the thermodynamic stability tests and show complete drug encapsulation. The droplet size of the NE system around 50 nm and PDI less than one has great significance, particularly for topical application. The same droplet size distribution has been widely reported for improved skin penetrability of loaded therapeutics after topical application [28,29]. NE6, NE7, and NE8 were selected for further investigation as they have droplet size around 50 nm with PDI less than one and negative zeta potential (as shown in Supplementary Figures S2–S4), which is the optimum droplet size and desirable surface charge reported for the topical application as it results in improved skin permeability and deeper penetration.

### 2.4. In Vitro Drug Release

**Figure 2:**
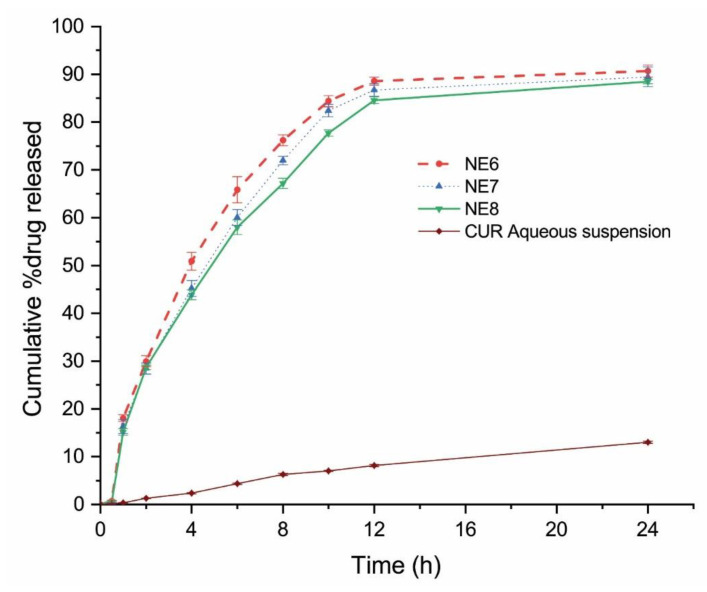
In vitro release of curcumin from curcumin-loaded NE (nanoemulsion) system.

Figure 2 shows the in vitro release behavior of curcumin from the screened formulation (NE6, NE7, and NE8) and curcumin aqueous suspension for 24 h. All investigated NE formulations were able to release 85% of curcumin within the first 12 h, while nearly 10% of curcumin was released from the curcumin aqueous suspension within the same time. This indicates that the droplet size of the NE system is an important determinant for in vitro drug release irrespective of the % composition of the NE system. This is in agreement with the previous investigation of Algahtani et al., which reported that curcumin NE (prepared by low energy emulsification technique with a higher concentration of Smix) of droplet size 70 nm with PDI 0.5 was able to release nearly 60% of curcumin within the first 12 h [12]. All the curcumin-loaded NE formulations showed a significantly (*p* < 0.05) higher release of curcumin compared to the in vitro release of curcumin from the aqueous suspension. Since the three tested formulations have similar release profiles, the selection of the formulation to be converted to NEG was based on the Smix concentration. Less Smix concentration in the topical formulation is more preferable when applied to an open wound. NE6 formulation was selected to be converted to NEG as it has the minimum concentration of Smix phase with maximum drug release rate (Figure 2).

### 2.5. Preparation and Characterization of Nanoemulgel

**Figure 3:**
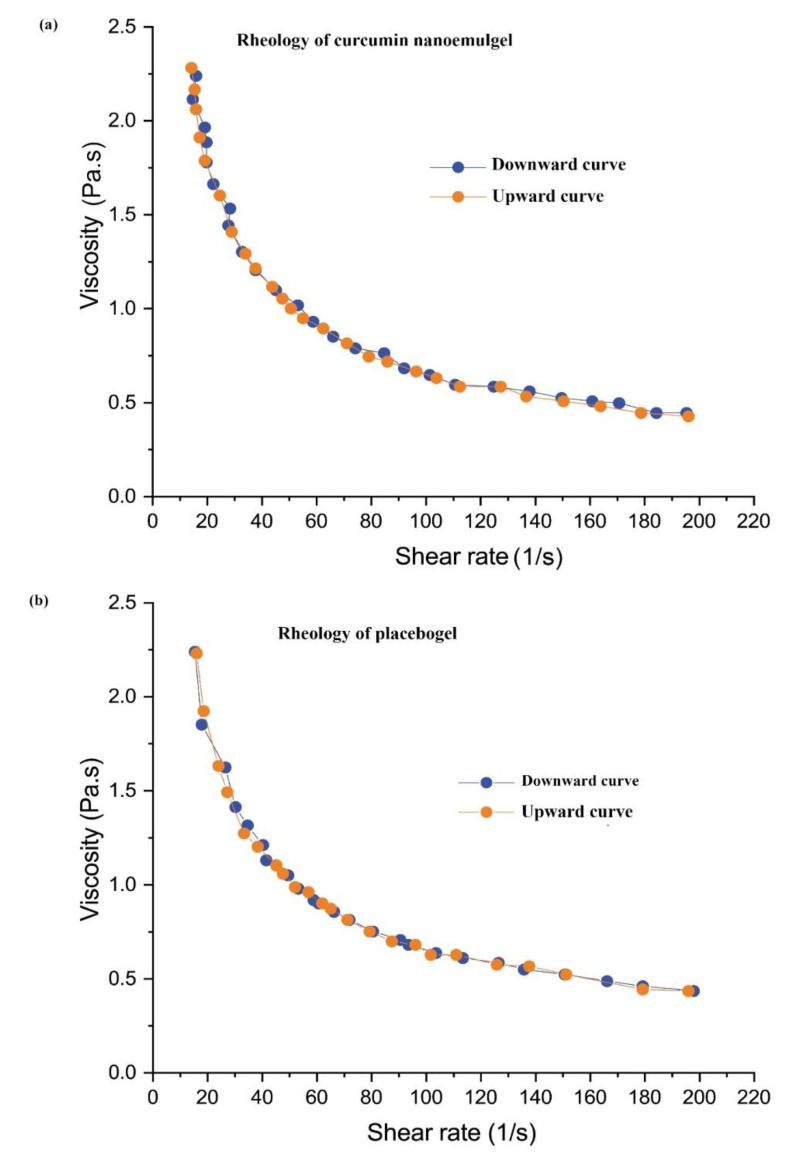
Rheology profile of (**a**) placebo gel and (**b**) Nanoemulgel of curcumin.

NE formulations in general have low viscosity and liquid in nature and specifically the curcumin NE has the addition of staining ability, which makes it difficult to be applied topically. Therefore, the NE6 was evenly dispersed in the Carbopol^®^ 940 gel matrix to achieve the final concentration of 0.5% (*w*/*w*) of curcumin into a developed NEG system with the desired consistency for patient-friendly topical application. The gel strength of curcumin NEG system (46.33 ± 1.154 s) and placebo gel system (44.66 ± 1.154 s) was measured and observed to be comparable to each other. The spreadability coefficient of curcumin NEG system and placebo gel system were determined and comparatively illustrated in Supplementary Figure S5. The pH range of the prepared curcumin NEG system was within the acceptable range of skin application and found close to the pH skin acid mantle (5.53 ± 0.03). This suggests that the developed formulation is safe to use for skin application, particularly for wound healing [20,23]. The % drug content uniformity of the curcumin NEG system was calculated. The formulation of curcumin NEG exhibited a uniform dispersion of curcumin in the hydrogel system with a uniformity of 98.93 ± 0.11%. The rheological profile of developed curcumin NEG and placebo gel of Carbopol^®^ 940 of the same concentration (0.5% *w*/*v*) are graphically shown in Figure 3a,b. The prepared curcumin NEG demonstrated a similar rheological profile as compared to placebo gel, and the incorporated curcumin NEG did not affect its rheology behavior. It is observed that the viscosity of placebo gel and curcumin NEG decreased upon an increase in applied shear rate and vice-versa (illustrated through downward and upward curves in Figure 3a,b). This property indicates the thixotropic behavior of the developed curcumin NEG system which is a desirable attribute for pharmaceutical dosage forms for topical application [30].

### 2.6. Ex-Vivo Skin Permeability Study

For drug permeability through the skin and its skin deposition, analysis from NEG and conventional gel of curcumin were carried with the help of Franz-diffusion cell. The results obtained from the ex-vivo skin permeability are shown in Table 3. The pH for curcumin-loaded NEG (5.53 ± 0.03) and conventional gel of curcumin (5.56 ± 0.02) was maintained with drug content uniformity of 98.93 ± 0.11 and 98.60 ± 0.52, respectively.

For drug permeation, the cumulative amount of curcumin permeated through the skin was 773.82 ± 1.08 from the curcumin NEG and 156.90 ± 0.95 µg/cm^2^ from the curcumin gel preparation. Whereas, the amount of curcumin deposited on the skin was 1161.54 ± 2.78 from the curcumin NEG and 179.47 ± 1.56 µg/cm^2^ from the curcumin gel preparation.

It is noteworthy to mention that there was an approximately six-fold increase of the percutaneous drug flux of curcumin from CUR-NEG (13.74 ± 1.08), compared to the drug flux of curcumin from the curcumin gel (2.19 ± 0.10). Likewise, the permeability coefficient (K × 10^−3^) of curcumin increased approximately six-fold from the curcumin NEG formulation (5.49 ± 0.67) when compared to the curcumin gel (0.876 ± 0.01). The permeation enhancement ratio (ER) of curcumin from the curcumin NEG was 6.27 ± 0.77, whereas the local accumulation efficiency (LAE) for curcumin was 1.50 ± 0.01 from the curcumin NEG and 1.14 ± 0.01 from the curcumin gel preparation.

The ex-vivo studies were performed to evaluate and compare the permeation and skin-deposition profile for the curcumin loaded in the two types of gel formulations. The high skin permeability of curcumin from the curcumin NEG system might be correlated to the encapsulation of curcumin inside the NE system. The encapsulation of loaded therapeutics inside nano oil droplets enhances its skin permeability profile and decreases the lag time for permeation [31]. The lag time (h) for curcumin released from the curcumin NEG was decreased to (0.75 ± 0.03) compared to the released curcumin from the conventional curcumin gel (2.37 ± 0.09). The enhanced skin-deposition of curcumin from curcumin NEG is imparted due to the use of Tween 80 in the Smix formulation as surfactant and it is known to enhance the dermal delivery of drugs [32]. Tween 80 modifies the skin barriers via readily entering the stratum corneum of the skin and showing a strong interaction with water within the cells, which affects the skin’s lipid and protein permeability, whereby the skin-permeation and deposition for the drugs are increased [33]. In this study, the high LAE value of curcumin released from the curcumin NEG is suggested to be related to the negatively charged surfaces of the drug-loaded oil droplets (Table 2) [34].

### 2.7. In-Vivo Wound Healing Activity

**Figure 4:**
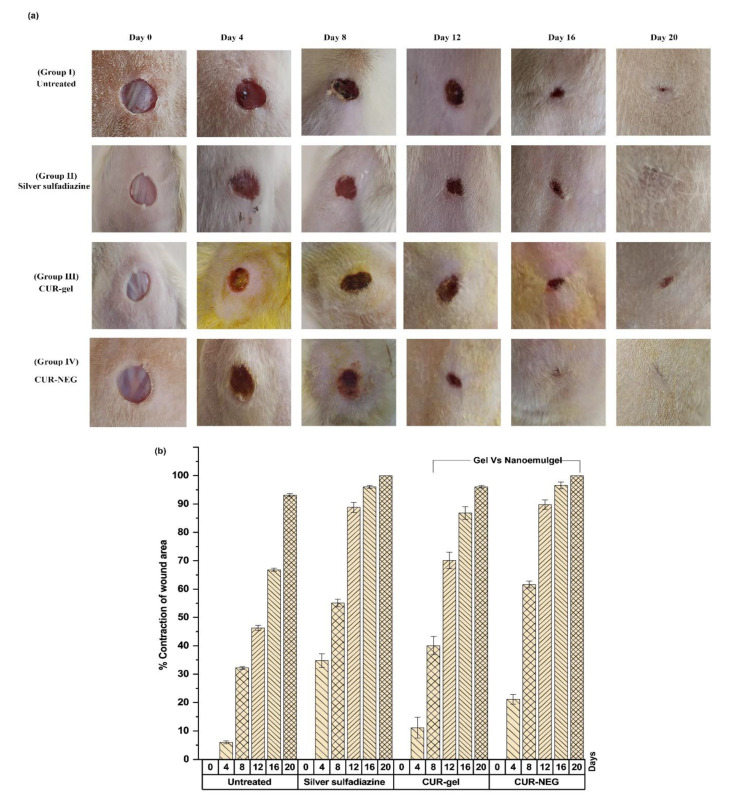
(**a**) In-vivo wound healing activity in Wistar rats (**b**) percentage contraction of wound area as an evaluation parameter for wound healing activity. Four groups were studied, Group I is the untreated group, Group II was treated with silver sulfadiazine cream, Group III was treated with the conventional curcumin gel (CUR-gel), and Group IV was treated with the curcumin NEG (CUR-NEG). Shaded bars represent the day number.

**Figure 5:**
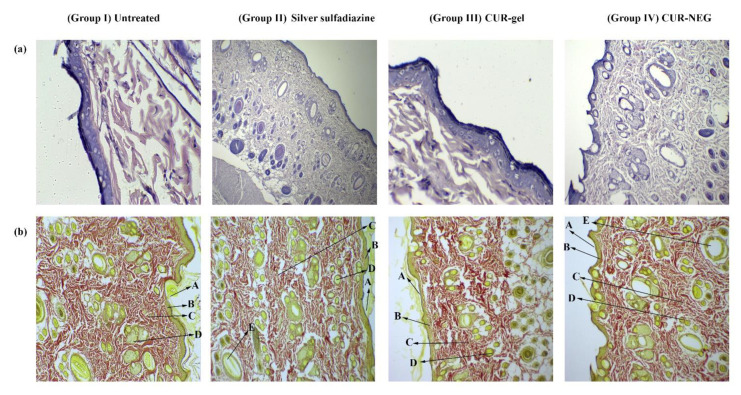
Histopathology analysis of newly healed tissue at day 20. (**a**) Stained with hematoxylin-eosin; (**b**) stained with vangeison to observe collagen formation (at 10× magnification). A—stratum corneum; B—papillary dermis; C—collagen fibers; D—sebaceous gland; E—hair follicles.

The wound healing activity of curcumin from the curcumin NEG and the conventional gel of curcumin were evaluated and compared to the commonly used and marketed formulation silver sulfadiazine (Figure 4). The topical applications of the studied formulations in Wistar rats were monitored for 20 days. Figure 5a, shows the appearance and the contraction of the wound at the days 0, 4, 8, 12, 16, and 20. Figure 5b shows the wound contraction percentage as the wound size at day zero was considered 100%.

Group I animals revealed a swelling with exudates on day four of post-wound observations (Figure 4a). The three treated groups showed a soft thrombus with a lack of discharge and reduced inflammation with a descending order of wound healing activity as Group IV > Group II > Group III. Moreover, the formation of brown-reddish tissues in the wound of G-I and III was observed on day eight; however, the formation of this structure was observed on day six for Group II and IV (not shown).

All the treated groups (Group II, III, IV) exhibited a remarkable wound healing activity compared to the untreated group (Group I), particularly the group treated with curcumin NEG (Group IV) and the group treated with silver sulfadiazine marketed cream (Group II) with almost complete wound contraction at the end of study i.e., day 20 (Figure 5a,b). For a proper epithelization of the observed wound, the untreated group required 16 days, whereas the time for epithelization observed in the three treated groups was as following: 14 days for the group treated with the curcumin gel (Group III), 11 days for the group treated with the silver sulfadiazine cream (Group II), and 10 days for the group treated with the curcumin NEG (Group IV).

Figure 4a,b denotes an almost equivalent wound healing activity for the animals treated with silver sulfadiazine marketed cream (standard drug) and with the curcumin NEG system. Curcumin is well-known for its wound healing activity [35,36], whereas, the formulation of curcumin in the form of NEG system further enhanced the wound healing activity of curcumin compared to the conventional gel formulation of curcumin.

Furthermore, the histopathological evaluation was performed to record the inflammation, collagen formation, and growth of the epithelial membrane. For this purpose, the Wistar rats’ skin from the tested groups (day 20) was subjected to histopathological procedures (Figure 5).

The comparative analysis revealed a high amount of granulomatous mass, fewer inflammatory cells, and extensive collagen fibers for the animals treated with curcumin NEG (Group IV) followed by the treated group with silver sulfadiazine marketed cream (Group II) and the group treated with the conventional gel of curcumin (Group III) (Figure 5). Additionally, the histopathological studies for the animal treated with curcumin NEG and silver sulfadiazine revealed the presence of papillary dermis with a thick epidermal layer, regeneration of sebaceous glands, as well as hair follicles with no sign of inflammation (Figure 5). The formation of granulation tissues, wound contraction, tissue remodeling, and collagen deposition properties for curcumin are already widely reported in the literature [8,9]. Indeed, the wound healing properties of loaded therapeutics along with incorporation of it into NEG as a delivery vehicle further imparts an auxiliary property to curcumin through deeper skin penetration, local deposition of drug in the skin, hence an augmented wound healing activity was observed for curcumin. The outcomes for in vivo healing process and histopathological examinations are in line with the previous investigation [17,24].

## 3. Conclusions

A nanoemulgel system with a uniform dispersion of curcumin was successfully designed and evaluated for ex vivo skin penetrability attributes along with in vivo wound healing efficacy in Wistar rats. The encapsulation of curcumin in O/W nanoemulsion of droplet size around 50 nm using a minimum concentration of surfactant is desirable for wound healing application and achieved effectively exploiting ultrasonic emulsification technique. This simple step product development process is of great significance for the industrial scalability of a nanoemulsion-based pharmaceutical product containing a therapeutic agent of poor biopharmaceutical attributes.

## 4. Materials and Methodology

### 4.1. Materials

Labrafac PG (Propylene glycol dicaprylocaprate) was obtained from Gattefose, France. Tween 80 (Polyoxyethylene sorbitan monooleate) was purchased from Sigma Aldrich, Germany. PEG 400 (Polyethylene glycol 400) was purchased from Merck, Schuchardh, Hokenbrunn, Germany. Water was obtained from the Milli-Q-water purification system (Millipore, Billerica, MA, USA). All the other excipients and chemicals were of pharmaceutical grade and/or analytical grade.

### 4.2. Screening of NE Components

The principle formulation components utilized to design an NE system for topical application prepared through low-energy/high-energy emulsification technique are oil, surfactant, and co-surfactant [20]. The oil phase of the NE system was screened based on the maximum amount of curcumin solubilize in it to achieve loading of therapeutic concentration. The solubility of the drug was also assessed in the surfactant/co-surfactant phase, which will also be helpful to predict the maximum loading of therapeutic concentration in the designed NE system. Briefly, excess amount of the drug in 2 mL of the selected vehicle in 5 mL capacity stoppered vials and mixed using a vortex mixer. These vials were then kept at 25 ± 2 °C in an isothermal water bath shaker for 48 h to equilibrate. The equilibrated samples were centrifuged at 5000× *rpm* for 15 min and supernatants were filtered through a syringe membrane filter (Whatman^®^ Puradisc, 0.22 µm) and the amount of solubilized drug was measured by UV-visible spectrophotometer at 425 nm [12].

Further, the emulsification efficiency of the surfactant and co-surfactant combination (Smix) was evaluated for screened oil phase. The emulsification efficiency of the Smix phase for screened oil phase was determined according to the previously reported method with slight modification [37]. Briefly, the oil-in-water (O/W) dispersion system was prepared through shearing with the addition of a known amount of oil phase (5 µL) to the 5% aqueous dispersion system of Smix, through a vortex mixture after each addition until the appearance of turbidity just developed. The percentage transmittance (%T) of the developed oil-in-water (O/W) dispersion system was determined through a UV-visible spectrophotometer at 683.2 nm [17]. The value of %T should be more than 80% to consider the endpoint of incorporation of oil phase into the 5% aqueous dispersion system of Smix phase. The aqueous dispersion system of Smix phase, having the ability to emulsify the desired amount of the screened oil phase, was allowed to equilibrate and visually observe to remain as a homogenous dispersion system.

The results obtained from the solubility of the drug in formulation components as well as emulsification efficiency of Smix phase for screened oil phase formed the basis to select the formulation components of the designed NE system.

### 4.3. Preparation of Curcumin NE through High-Energy Emulsification

The high-energy ultrasonication technique was utilized to prepare the curcumin-loaded NE system [18]. Briefly, the O/W aqueous dispersion system consists of 1% *w*/*w* (10 mg/g) of curcumin in the mixture of the oil and Smix phase through vortex mixture followed by addition of the aqueous phase with continuous vortexing for 1 min [15]. The generated O/W aqueous dispersion system was ultrasonicated (Ultrasonic Homogenizer, FS-300N, Zhejiang, China; 300 W with continuous adjustable ultrasonication amplitude 0–100%) further in a water bath for different periods (3 and 5 min) at a constant ultrasonication amplitude of 40% (power 120 W) [17,19]. Eight NE formulations of different compositions were designed and developed for further investigation for droplet size and polydispersity index (PDI) to select the desirable formulation of the curcumin-loaded NE system.

### 4.4. Characterization of Curcumin NE

Initially, curcumin-loaded NE formulations were prepared in triplicate by high-energy ultrasonication technique and formulation of desirable attributes were evaluated for thermodynamic stability, droplet size distribution, PDI, zeta potential, viscosity, and analysis of % drug content. These experiments were carried out in triplicate.

#### 4.4.1. Thermodynamic Stability

The prepared curcumin loaded-NE system was subjected to different stress conditions tests such as heating–cooling cycles (4 and 40 °C) and freeze–thaw cycles (−21 and +25 °C) with storage at specified temperature for 48 h. For centrifugation stress study, 1 mL of the curcumin-loaded NE system was diluted to 100 mL with distilled water and centrifuged at 5000× *rpm* for 30 min, and visually observed for any phase separation [21].

#### 4.4.2. Droplet Size, Polydispersity Index, and Zeta Potential

The average droplet size and PDI of different compositions of curcumin-loaded NE system were analyzed at 25 °C by Dynamic Light Scattering (DLS) technique using a Zetasizer Nano ZS90 (Malvern Instruments, Malvern, UK) [22]. The zeta potential of the curcumin-loaded NE system was also determined using the same instrument.

#### 4.4.3. Determination of Curcumin NE Viscosity

The viscosity of the optimized curcumin-loaded NE system was analyzed, without dilution, using a Bohlin rotational viscometer at ambient temperature (25 °C) [19,21].

#### 4.4.4. Analysis of the Drug Content

The % content of curcumin in the screened NE formulations was determined by a UV-visible spectrophotometer at λmax 425 nm [12]. A 100 µL sample of curcumin-loaded NE was diluted to 1000 times with methanol.

### 4.5. In-Vitro Drug Release

The curcumin-loaded NE system, which passes the thermodynamic stability and has a droplet size near 50 nm (NE6, NE7, and NE8), was chosen for the in vitro drug release investigation through the dialysis bag technique [38]. Dialysis bags (Merck dialysis sacks, 12–14 kDa) were filled with 1 mL of curcumin-loaded NE formulation and suspended in phosphate buffer release medium of pH 7.4 and maintained at 37 ± 0.5 °C. At a defined interval of time, 1 mL aliquots were taken out and replaced by the same volume of release medium. The amount of curcumin in the aliquots was quantified by UV-spectroscopy at λmax 425 nm. These experiments were carried out in triplicate.

### 4.6. Preparation and Characterization of Curcumin Nanoemulgel

Curcumin NEG system was prepared by dispersing the optimized formulation (NE6) in Carbopol^®^ 940 (0.5% *w*/*w*) gel [12]. Glycerin (5% *w*/*w*) was added as a humectant to the dispersion system to provide a smooth and soothing effect [20]. Triethanolamine was added into the dispersion system drop-by-drop to neutralize the pH to 5.5, resulting in instant conversion to a hydrogel system [17]. The pH, rheology, and drug content uniformity of the curcumin in the NEG system were evaluated by the method previously reported by our group [17,20].

### 4.7. Ex-Vivo Skin Permeability

The ex-vivo skin permeability profile of the developed curcumin-loaded NEG and conventional gel of curcumin were performed as per the method described previously [17,20]. The excised skin from the Wistar rat was utilized for the ex vivo skin permeation experiment. Local accumulation efficiency (LAE) of the curcumin in NEG and conventional gel were obtained as a ratio of curcumin accumulated in the skin to that permeated through the skin [38,39].

### 4.8. In-Vivo Study

#### 4.8.1. Experimental Protocol

The animal protocol to carry out the in-vivo wound healing activity and histopathological examination were approved by the institutional ethical committee (Najran University, KSA) and followed their guidelines to perform the studies (Ref. No: 25-01-02-20-EC). Albino rats (weight 200–250 g) were used for the study. The animals were kept under standard laboratory conditions (temperature: 25 ± 2 °C; relative humidity: 55 ± 5%). The animals were housed in polypropylene cages, with free access to a standard laboratory diet and water ad libitum. Animals were anesthetized under aseptic conditions, using 50 mg/kg Ketamine HCl intraperitoneally [40]. Animals were placed on a level surface, their back was shaved, and a deep wound area was created using a sterile 8 mm biopsy punch (Acu punch, Acuderma Inc, Louderale, FL, USA).

All animals were divided into four groups with four rats in each group. Group I is the negative control group with no treatment, group II is the treated group with the marketed preparation of 1% *w*/*w* silver sulfadiazine cream twice a day, group III is the group treated with 0.5% curcumin-gel twice a day, and group IV is the group treated with 0.5% curcumin NEG twice a day. All the treated groups (Group II, III, and IV) received the therapy for 20 consecutive days.

#### 4.8.2. Evaluation of Wound Healing Area

Evaluation of wound healing area was performed in terms of wound contraction percentage, wound closure time, and epithelialization period [41,42]. Percentage of wound contraction was calculated taking the initial size of the wound as 100% using the following formula.

(1) $$\%wound\;contraction=\frac{\left(Initial\;wound\;area-Specific\;day\;wound\;area\right)}{Initial\;day\;wound\;area}\times 100$$

#### 4.8.3. Histopathology

On the last day of the wound healing experiment, the animals were anesthetized using Ketamine HCl (50 mg/kg, i.p.), euthanized, and specimens of wound tissue with the adjacent healthy tissue were collected. The collected samples were fixed in 10% formalin and were subjected to routine histopathological tissue examination [17]. The wound tissue specimen was sectioned with a microtome (Leica RM 2245) and then stained with hematoxylin-eosin. The prepared tissue slide was examined under a light microscope. Further, to evaluate the collagen content, the wound tissue specimen was sectioned using a microtome, stained with Van Gieson stain for collagen fiber, and examined under a microscope (Leica DM IRM, Leica Microsystems, Wetzlar, Germany).

### 4.9. Statistical Analysis

The statistical analysis was performed using SPSS (version 23 SPSS Inc., Chicago, IL, USA). The obtained data were analyzed utilizing one-way ANOVA followed by Tukey’s multiple comparisons tests, where *p* < 0.05 was considered statistically significant.

## Figures and Tables

**Table 1:** Formulation composition of curcumin loaded NE system prepared through ultrasonication technique.

Formulation Code	Oil%	Smix%	Water%	Smix Ratio	Ultrasonication Time (Min)	Mean Droplet Size (Nm)	Polydispersity Index
NE1	20.0	25.0	55.0	1:1	3.0	232.80 ± 11.04	0.23 ± 0.16
NE2	20.0	25.0	55.0	1:1	5.0	133.76 ± 2.84	0.29 ± 0.11
NE3	20.0	30.0	50.0	1:1	3.0	152.70 ± 3.08	0.20 ± 0.04
NE4	20.0	30.0	50.0	1:1	5.0	116.63 ± 2.22	0.14 ± 0.12
NE5	20.0	25.0	55.0	2:1	3.0	84.23 ± 1.33	0.23 ± 0.05
NE6	20.0	25.0	55.0	2:1	5.0	56.25 ± 0.69	0.05 ± 0.01
NE7	20.0	30.0	50.0	2:1	3.0	51.47 ± 0.71	0.13 ± 0.04
NE8	20.0	30.0	50.0	2:1	5.0	49.61 ± 0.53	0.10 ± 0.01

**Table 2:** Characterization of selected CUR-loaded NE for thermodynamic stability, droplet size distribution, zeta potential, % drug content, and viscosity.

Formulation Code	Thermodynamic Stability			Mean Droplet Size (nm)	Polydis-persity Index	Zeta Potential (mV)	Drug Content (%)	Viscosity (mPas)
	Heating Cooling Cycle	Centrifugation Study	Freeze-Thaw Cycle					
NE5	√	√	√	84.23 ± 1.33	0.23 ± 0.05	−15.96 ± 0.55	98.86 ± 0.58	89.82 ± 1.27
NE6	√	√	√	56.25 ± 0.69	0.05 ± 0.01	−20.26 ± 0.65	99.23 ± 0.28	83.74 ± 1.92
NE7	√	√	√	51.47 ± 0.71	0.13 ± 0.04	−19.26 ± 0.20	99.16 ± 0.15	85.05 ± 2.30
NE8	√	√	√	49.61 ± 0.53	0.10 ± 0.01	−19.6 ± 0.41	99.03 ± 0.41	87.37 ± 0.66

**Table 3:** Characterization of curcumin-loaded nanoemulgel to determine skin penetrability profile compared to conventional curcumin gel. “ER—enhancement ratio”.

Formulation	Cumulative Amount of Drug Permeated (µg/cm^2^)	Drug Deposited in Skin (µg/cm^2^)	Lag Time (h)	Flux (µg/cm^2^.h)	Permeability Coefficient (K × 10^−3^)	ER	Local Accumulation Efficiency (LAE)
CUR-NEG	773.82 ± 1.08	1161.54 ± 2.78	0.75 ± 0.03	13.74 ± 1.08	5.49 ± 0.67	6.27 ± 0.77	1.50 ± 0.01
CUR-gel	156.90 ± 0.95	179.47 ± 1.56	2.37 ± 0.09	2.19 ± 0.10	0.876 ± 0.01	-	1.14 ± 0.01

## Data Availability

The data presented in this study are available in article or Supplementary Materials.

## Supplementary Materials

The following are available online at https://www.mdpi.com/article/10.3390/gels7040213/s1, Figure S1: Volume of optimized oil phase (Labrafac PG) emulsified in Smix phase (Tween 80 and PEG 400) at different ratios (1:1, 2:1, 3:1, and 4:1) to measure its emulsification efficiency and corresponding value of the percentage transmittance., Figure S2: Droplet size (56.64 nm with PDI 0.054) and zeta potential (−20.3 mV) of NE6., Figure S3: Droplet size (51.10 nm with PDI 0.181) and zeta potential (−18.8 mV) of NE7, Figure S4: Droplet size (49.39 nm with PDI 0.113) and zeta potential (−18.1 mV) of NE8., Figure S5: Spreadability behavior of curcumin NEG system and placebo gel system.
