# Supplementary material for: Preparation and Characterization of Curcumin Nanoemulgel Utilizing Ultrasonication Technique for Wound Healing: In Vitro, Ex Vivo, and In Vivo Evaluation
Source: Gels. 2021 Nov 14;7(4):213. doi: 10.3390/gels7040213 (PMC8628781; doi:10.3390/gels7040213)
Supplement: Supplementary file 1 [file gels-07-00213-s001.zip › gels-1443237-SI.pdf]

gels

Article

Preparation and Characterization of Curcumin Nanoemulgel Utilizing Ultrasonication Technique for Wound Healing: In Vitro, Ex Vivo, and In Vivo Evaluation

Mohammed S. Algahtani [1], Mohammad Zaki Ahmad [1], Ihab Hamed Nourein [2], Hassan A. Albarqi [1], Hamad S. Alyami [1], Mohammad H. Alyami [1], Abdulsalam A. Alqahtani [1], Ali Alasiri [1], Thamer S. Algahtani, Abdul Aleem Mohammed [1] and Javed Ahmad [1,*]

[1] Department of Pharmaceutics, College of Pharmacy, Najran University, Najran 11001, Saudi Arabia; msalqahtane@nu.edu.sa (M.S.A.); mzahmad@nu.edu.sa (M.Z.A.); haalbarqi@nu.edu.sa (H.A.A.); hsalmukalas@nu.edu.sa (H.S.A.); mhalmansour@nu.edu.sa (M.H.A.); aamari@nu.edu.sa (A.A.A.); aalasiri@nu.edu.sa (A.A.); Abdul Aleem Mohammed (A.A.M.)

[2] Department of Clinical Laboratory (Histopathology and Cytology), College of Applied Medical Sciences, Najran University, Najran 11001, Saudi Arabia; ihab213@gmail.com

* Correspondence: jaahmed@nu.edu.sa; Tel.: +966-17542-8744

Supplementary Materials

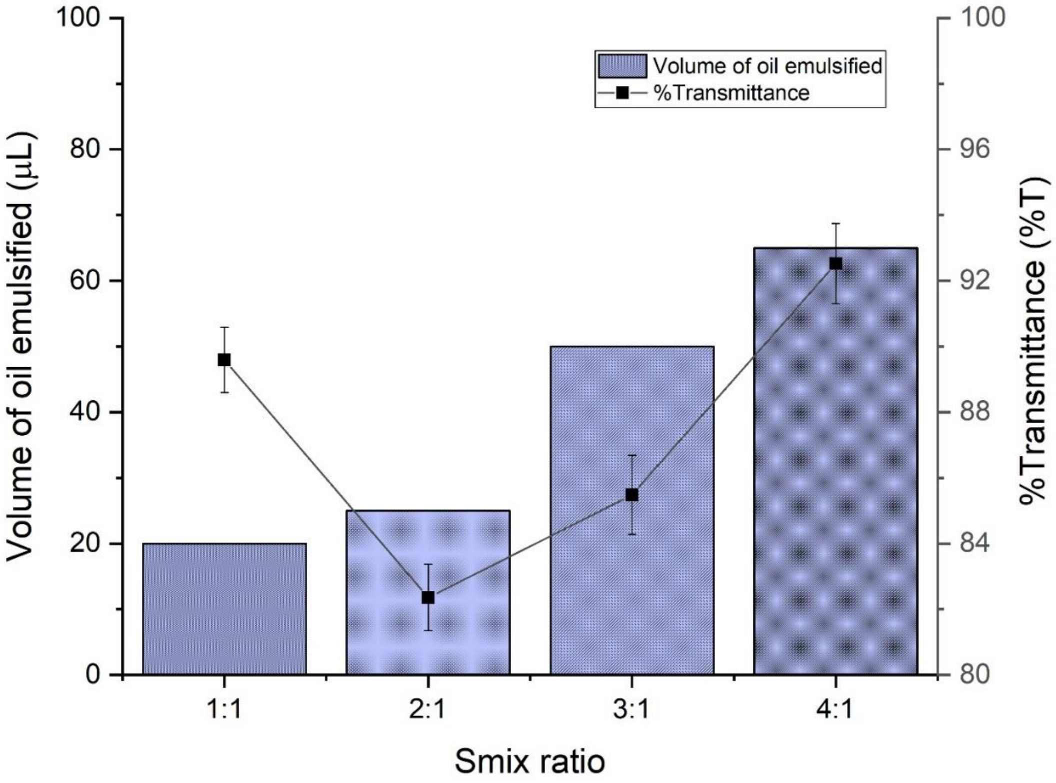

Figure S1. Volume of optimized oil phase (Labrafac PG) emulsified in Smix phase (Tween 80 and PEG 400) at different ratios (1:1, 2:1, 3:1, and 4:1) to measure its emulsification efficiency and corresponding value of the percentage transmittance.

Results

			Diam. (nm)	% Intensity	Width (nm)
Z-Average (r.nm):	56.64	Peak 1:	60.77	100.0	16.77
PdI:	0.054	Peak 2:	0.000	0.0	0.000
Intercept:	0.934	Peak 3:	0.000	0.0	0.000
Result quality :	Good				

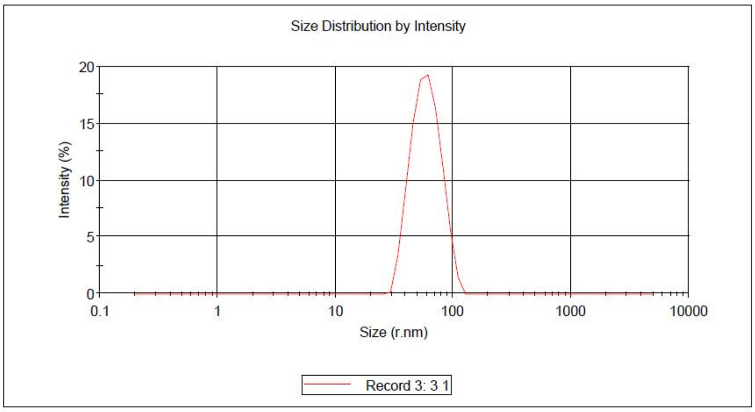

Results

			Mean (mV)	Area (%)	Width (mV)
Zeta Potential (mV):	-20.3	Peak 1:	-20.3	100.0	5.39
Zeta Deviation (mV):	5.39	Peak 2:	0.00	0.0	0.00
Conductivity (mS/cm):	0.00570	Peak 3:	0.00	0.0	0.00
Result quality :	Good				

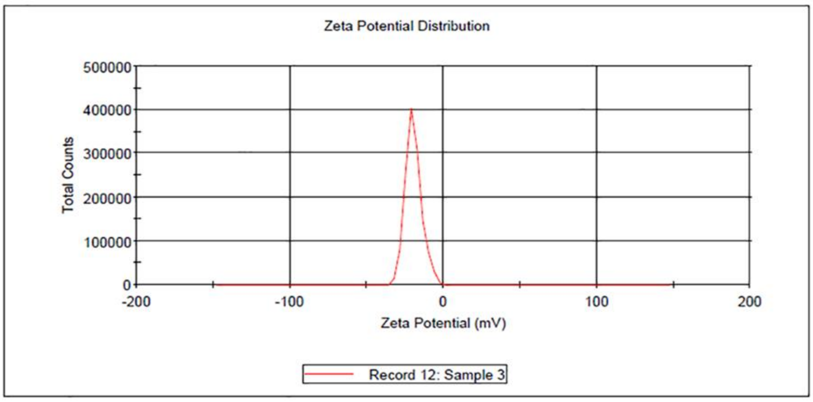

Figure S2. Droplet size (56.64 nm with PDI 0.054) and zeta potential (-20.3 mV) of NE6.

Results

			Diam. (nm)	% Intensity	Width (nm)
Z-Average (r.nm):	51.10	Peak 1:	63.58	100.0	30.61
PdI:	0.181	Peak 2:	0.000	0.0	0.000
Intercept:	0.923	Peak 3:	0.000	0.0	0.000
Result quality :	Good				

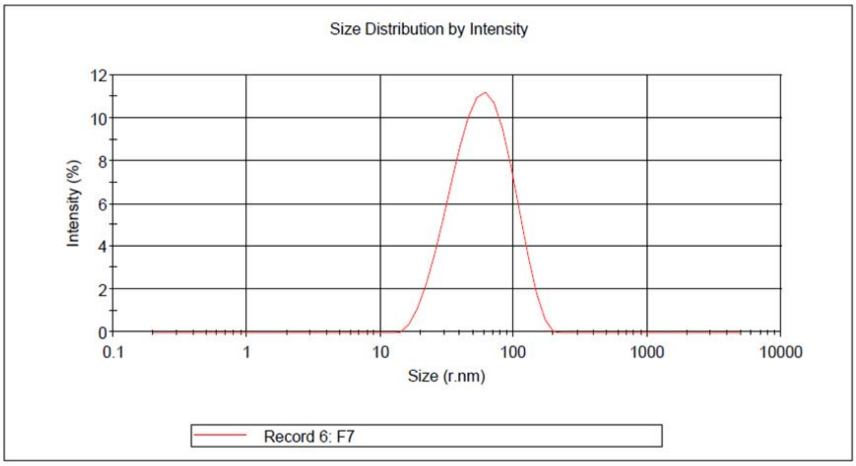

Results

			Mean (mV)	Area (%)	Width (mV)
Zeta Potential (mV):	-18.8	Peak 1:	-18.8	100.0	5.24
Zeta Deviation (mV):	5.24	Peak 2:	0.00	0.0	0.00
Conductivity (mS/cm):	0.00584	Peak 3:	0.00	0.0	0.00
Result quality :	Good				

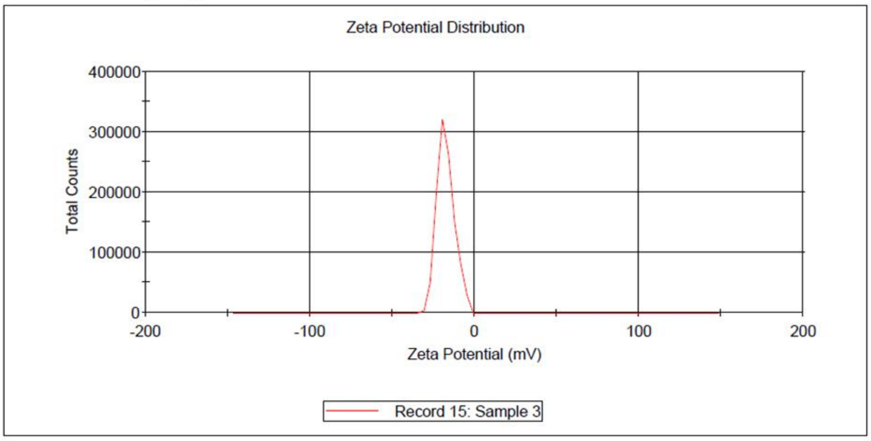

Figure S3. Droplet size (51.10 nm with PDI 0.181) and zeta potential (-18.8 mV) of NE7.

Results

			Diam. (nm)	% Intensity	Width (nm)
Z-Average (r.nm):	49.39	Peak 1:	56.37	100.0	21.25
PdI:	0.113	Peak 2:	0.000	0.0	0.000
Intercept:	0.921	Peak 3:	0.000	0.0	0.000
Result quality :	Good				

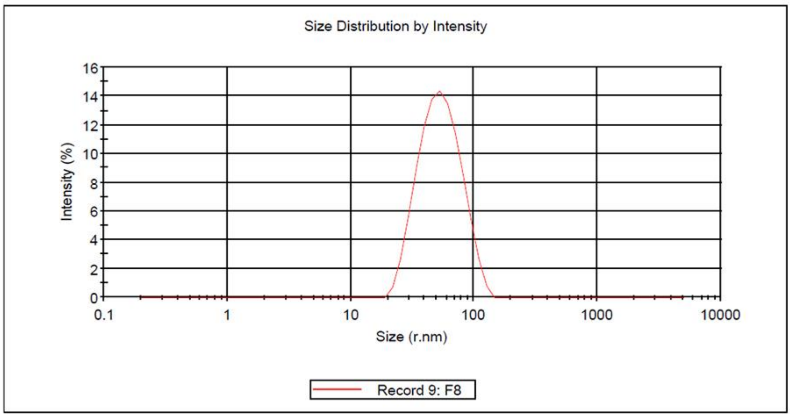

Results

			Mean (mV)	Area (%)	Width (mV)
Zeta Potential (mV):	-18.1	Peak 1:	-18.1	100.0	4.84
Zeta Deviation (mV):	4.84	Peak 2:	0.00	0.0	0.00
Conductivity (mS/cm):	0.0101	Peak 3:	0.00	0.0	0.00
Result quality :	Good				

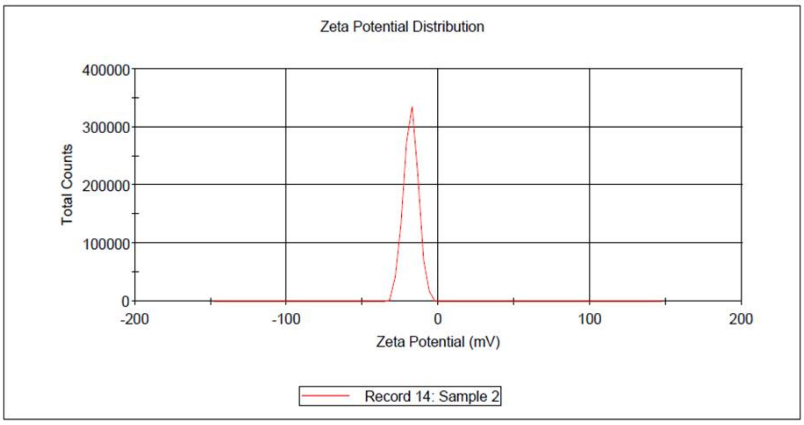

Figure S4. Droplet size (49.39 nm with PDI 0.113) and zeta potential (-18.1 mV) of NE8.

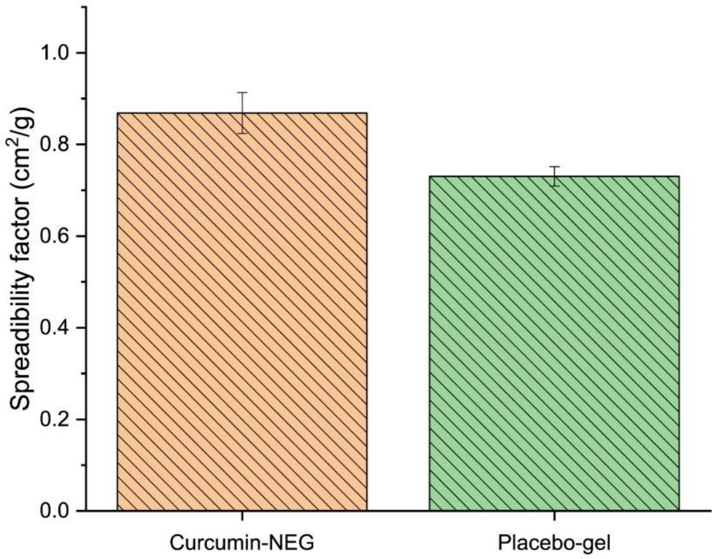

Figure S5. Spreadability behavior of curcumin NEG system and placebo gel system.
